# Characterization of suprathermal electrons inside a laser accelerated plasma via highly-resolved K_⍺_-emission

**DOI:** 10.1038/s41467-019-12008-9

**Published:** 2019-09-16

**Authors:** M. Šmíd, O. Renner, A. Colaitis, V. T. Tikhonchuk, T. Schlegel, F. B. Rosmej

**Affiliations:** 1 Helmholtz-Zentrum Dresden-Rossendorf, 01328 Dresden, Germany; 2 Institute of Physics of the Czech Academy of Sciences, 18221 Prague, Czech Republic; 3 ELI Beamlines, Institute of Physics of the Czech Academy of Sciences, 25241 Dolni Brezany, Czech Republic; 4 Institute of Plasma Physics of the Czech Academy of Sciences, 18200 Prague, Czech Republic; 5 Centre Lasers Intenses et Applications, University of Bordeaux - CNRS - CEA, 33405 Talence, France; 6 Sorbonne Université, Faculté des Sciences et Ingénierie, UMR7605, F-75252 Paris, France; 7 LULI, École Polytechnique, CEA, CNRS, Atomic Physics in Dense Plasmas PAPD, F-91128 Palaiseau, France; 8 Moscow Institute of Physics and Technology - MIPT, Dolgoprudnyi, 141700 Russia; 9 National Research Nuclear University - MEPhI, Department of Plasma Physics, Moscow, 115409 Russia

**Keywords:** Atomic and molecular physics, Laser-produced plasmas, Characterization and analytical techniques

## Abstract

Suprathermal electrons are routinely generated in high-intensity laser produced plasmas via instabilities driven by non-linear laser-plasma interaction. Their accurate characterization is crucial for the performance of inertial confinement fusion as well as for performing experiments in laboratory astrophysics and in general high-energy-density physics. Here, we present studies of non-thermal atomic states excited by suprathermal electrons in kJ-ns-laser produced plasmas. Highly spatially and spectrally resolved X-ray emission from the laser-deflected part of the warm dense Cu foil visualized the hot electrons. A multi-scale two-dimensional hydrodynamic simulation including non-linear laser-plasma interactions and hot electron propagation has provided an input for ab initio non-thermal atomic simulations. The analysis revealed a significant delay between the maximum of laser pulse and presence of suprathermal electrons. Agreement between spectroscopic signatures and simulations demonstrates that combination of advanced high-resolution X-ray spectroscopy and non-thermal atomic physics offers a promising method to characterize suprathermal electrons inside the solid density matter.

## Abstract (web summary)

Suprathermal electrons in laser-generated plasmas are potentially useful in many plasma environments. Here the authors show the characterization of suprathermal electrons in laser-generated Cu plasma using a high-resolution X-ray spectroscopy combined with hydrodynamic and atomic modeling.

## Introduction

Hot (suprathermal) electron (HE) production driven by instabilities in laser plasma interaction^1^ is of paramount interest for the inertial confinement fusion (ICF) science and high-energy-density-physics^2–5^. Hot electrons can cause degradation in the performance of ICF capsules by fuel preheat and reduced compressibility of the capsule^2,6,7^. In the fast ignition scheme^8^, laser coupling to fast electrons^9^ and their subsequent transport^10,11^ determine the efficiency of the energy delivery to the ignition region.

In the shock ignition scheme^12^ the fuel is ignited from a central hot spot heated by a strong spherically convergent shock. The laser intensities required to launch this shock exceed the threshold of parametric instabilities (such as stimulated Raman scattering (SRS) or two–plasmon decay), which couple a significant fraction of the laser energy to hot electrons^13,14^.

Shock ignition experiments in the planar geometry reported pressures up to $90\ {\rm{Mbar}}$^15–17^. In spherical geometry experiments, the shock strength was shown to be significantly enhanced by the generation of hot electrons; their total energy reached up to $8 \%$ of the incident laser energy and the shock pressure exceeded 300 Mbar^18,19^.

Hydrodynamic simulations of laser-plasma interactions for pulse durations of the order of $0.1-10\ {\rm{ns}}$ and irradiations $I{\lambda }^{2}=1{0}^{13}-1{0}^{16}\ {\rm{W}}{\rm{cm}^{-2}}\ \upmu {\rm{m}}^{2}$ are highly challenging as non-linear processes play an important role. These kinetic processes cannot be directly incorporated into large scale hydrodynamic models because of a strong disparity of temporal and spatial scales. A detailed characterization of hot electrons as well as the plasma evolution via independent methods is therefore mandatory to validate large scale hydrodynamic simulations. In this context, X–ray spectroscopy and non–thermal atomic physics are of particular interest due to their potential for a unique characterization of hot electrons^20–26^. Moreover, optimized ${\rm{K}}_\alpha$-emission X–ray sources have also been employed for scattering and backlighting^27,28^.

Non-Maxwellian atomic population kinetics has been applied to quantitatively determine the fraction of hot electrons in hohlraums and to identify relevant parametric instabilities^20,24^ whereas ${\rm{K}}_{\alpha}$-emission from sandwich geometries provided a time integrated information on the hot electron temperature, absolute flux, and spatial distribution^17,29,30^. The problem of detection of the temporal evolution of hot electrons inside near solid density matter with high-resolution X-ray spectroscopy (where contributions to ${\rm{K}}_{\alpha }$-emission from different charge states are spectrally and spatially resolved) have not yet been addressed. This, however, is an important issue for providing experimental reference data for advanced kinetic simulations of non-linear laser plasma interaction. Moreover, the timing of hot electron generation becomes of increasing importance for the shock ignition scheme as recent experiments have demonstrated that hot electron onset has a strong influence on the shock strength^31^.

In this paper we address critical timing issues of hot electron generation and propagation inside near solid density matter via state-of-the-art high-resolution X-ray spectroscopy^34,35^ and large-scale non-linear 2D-hydro-simulations^36^. The particular link between hydrodynamic simulations, non-thermal atomic kinetics and highly-resolved (in frequency and space) X-ray emission spectroscopic data can also be understood as a benchmark for the implementation of non-linear laser-plasma interaction in large-scale hydrodynamic simulations.

## Results

### Spatial offset in measured spectra

**Fig. 1:**
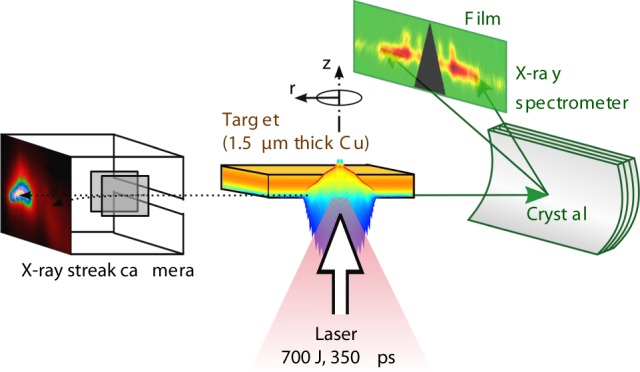
Experimental setup. The laser beam ($\lambda =1.315\ \upmu {\rm{m}}$, $I=4.55\times 1{0}^{15}\ {\rm{W}}{{\rm{cm}}}^{-2}$) irradiates a 1.5–$\upmu {\rm{m}}$–thick Cu foil at normal incidence. The Cu K–shell emission is observed by spatially resolving crystal spectrometer and a temporally resolving streak camera

**Fig. 2:**
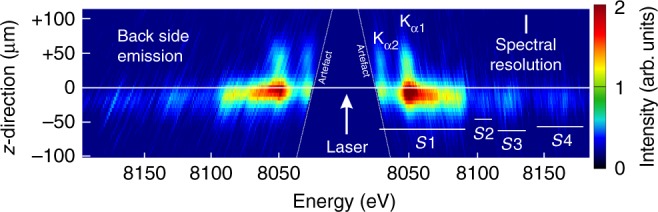
Experimental spectrum. Mirror-symmetric spectral records show ${\rm{K}}_{\alpha}$-structure (cold ${\rm{K}}_{\alpha,1,2}$ as well as heated ${\rm{K}}_{\alpha}$-emission of groups $S1,S2,S3,S4$) and its extension along $z$-axis. The spectral resolution of $\lambda /\Delta \lambda \approx 5200$ is visualized by the width of the white vertical bar ($\approx 1.5\ {\rm{eV}}$)

The spatially and spectrally resolved X-ray emission from Cu foil irradiated by kJ-ns-laser (cf. the schematic experimental configuration presented in Fig. 1) is shown in Fig. 2. The ${\rm{K}}_{\alpha}$-satellites above photon energy of 8070 eV are observed only in a region close to the original target position ($z=0$) and below it. The ${\rm{K}}_{\alpha 1,2}$ –lines (8047 and 8027 eV), however, are extended up to $z\ge 50\ \upmu {\rm{m}}$. This emission is originating from the cold foil material and witnesses the deformation of the Cu foil during the laser impact: As the hot electrons are penetrating into the accelerated foil, they cause X-ray material fluorescence and therefore visualize the plasma displacement in positive $z$-direction.

**Fig. 3:**
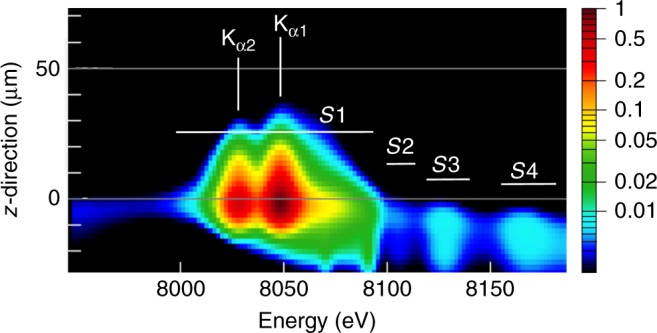
Synthetic $z$-resolved spectra. The spectral intensities are mapped into colors on logarithmic scale

**Fig. 4:**
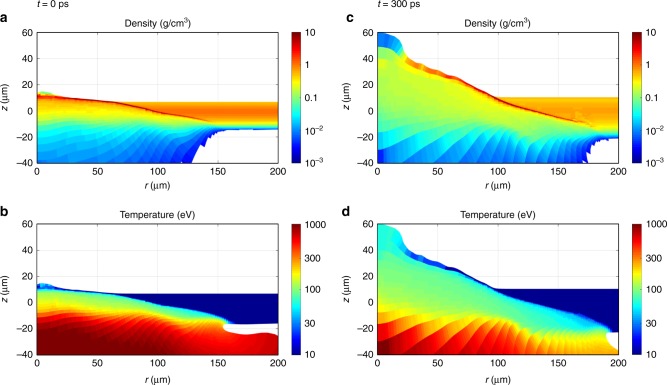
Plasma profiles from the hydrodynamic simulation. 2D distributions ($r,z$) of the plasma mass density (**a**, **c**) and temperature (**b**, **d**) on logarithmic scale obtained from the hydrodynamic simulation at the time of the laser pulse maximum ($t=0$) (a, **b**) and at the end of the laser pulse, $t=300\ {\rm{ps}}$ (**c**, d)

In order to compare the experimental data with complex hydrodynamic and atomic simulations, the time- and space-dependent synthetic spectra were temporally and radially integrated, but spatially resolved in $z$-direction, as shown in Fig. 3, so that they can be directly compared with the experimental data (Fig. 2). The synthetic data show the ${\rm{K}}_{\alpha 1,2}$-emission extended up to approximately $35\ \upmu {\rm{m}}$ beyond the original target position (positive $z$-values). This extension is explained by the hydrodynamic motion of the cold foil material under the laser-induced ablation pressure. The ${\rm{K}}_{\alpha}$-emission is originating from the thin layer of dense and cold material, as seen in the plots of spatially resolved plasma parameters calculated by the hydrodynamic code (red and blue colors in panels b and d, respectively) in Fig. 4. The extension of the ${\rm{K}}_{\alpha 1,2}$-emission in forward direction (positive $z$-values) is in good agreement with the measured data that show emission extension up to $50\ \upmu {\rm{m}}$ (Fig. 2). These findings are further supported by streak camera measurements (see Methods). Agreement in the spatial extent of the cold ${\rm{K}}_\alpha$-lines indicates that the laser induced foil acceleration is well described.

The ${\rm{K}}_{\alpha 1,2}$-emission shows rather broad lines in contrast with narrow transitions emitted from an X-ray tube. This is related to the fact that dense material (red color in density profile of Fig. 4c) is heated up to temperatures of about 10–100 eV (Fig. 4d). For such temperatures, the wavelengths of ${\rm{K}}_{\alpha 1,2}$-emission of singly ionized copper depend rather weakly on the copper ionization because angular coupling and configuration interaction result in negative screening for the open $3d$ shell electrons. Therefore, the observed ${\rm{K}}_{\alpha 1,2}$-lines present an overlap of ${\rm{K}}_{\alpha}$-emission of many charge states that results in a noticeable broadening of both, ${\rm{K}}_{\alpha 1}$- and ${\rm{K}}_{\alpha 2}$-lines. The broadening is well observed in Fig. 2 thanks to a spectral resolution better than the width of the ${\rm{K}}_{\alpha 1}$- and ${\rm{K}}_{\alpha 2}$-lines. This observation indicates that temperatures at that stage of plasma expansion are well described in the simulations.

The data in Fig. 2 show also intense ${\rm{K}}_{\alpha}$-emission shifted to higher energies (groups *S*2, *S*3, *S*4), which originates from higher charge states. The first emission group *S*1 corresponds to open M-shell configurations, higher energy emission groups $S2,S3,S4$ correspond to open L-shell configurations. A much higher intensity of the emission group $S1$ compared to the groups $S2,S3,S4$ is well reproduced in the simulations. That observation demonstrates that accounting for the temporal evolution of hot electron population from the simulations, the observed spectroscopic features are well described.

### Temporal delay of suprathermal electron production

The simultaneously achieved high-spectral and high-spatial resolution allows also a temporal reconstruction of the suprathermal electron production from a time integrated original data set. As can be seen from the X-ray images (Fig. 2), ${\rm{K}}_{\alpha}$-emission of ${{\rm{Cu}}}^{+1}$ up to about ${{\rm{Cu}}}^{+15}$ (group *S*1) extends up to about $50\ \upmu {\rm{m}}$ beyond the original target position. These low charge states correspond to a plasma temperature <100 eV; higher temperature plasma would produce a more shifted ${\rm{K}}_{\alpha}$-emission. For low temperatures, however, thermal excitation of ${\rm{K}}_{\alpha}$-transitions is impossible because of a large disparity between the transition energy and the plasma temperature: only hot electrons penetrating into cold matter can produce such an effective emission. The emission induced by a several-hundreds eV electrons is exponentially smaller (see Eq. (2) and related discussion). An average expansion velocity of the foil can be derived from the simulations presented in Fig. 4. The cold part of the foil is displaced forward (in the direction to the laser) to about $35\ \upmu {\rm{m}}$ in 300 ps corresponding to an average velocity of $1.2\times 1{0}^{7}\ {\rm{cm}}/{\rm{s}}$. This value is in good agreement with the streak camera measurements of the foil emission displacement of $1-1.3\times 1{0}^{7}\ {\rm{cm}}/{\rm{s}}$. In order to produce such emission on the back side of the deflected foil, the hot electrons must be generated about 300 ps after the laser pulse maximum (the life time of hot electrons in a hot dense plasma is a few ps, much shorter than our temporal resolution). Observation of the retarded ${\rm{K}}_\alpha$-emission witnesses therefore a significant delay in the hot electron generation.

**Fig. 5:**
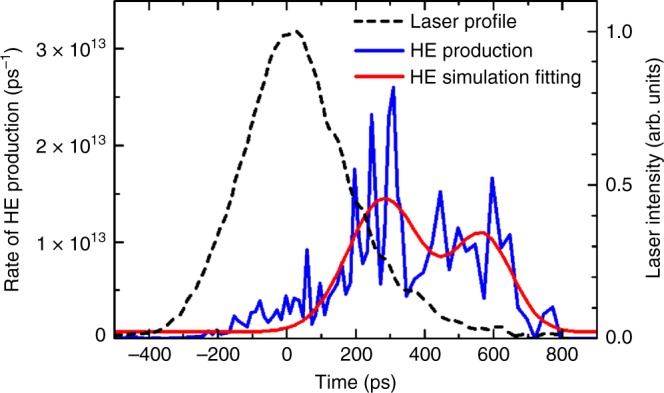
Temporal evolution of hot electrons. The production rate of hot electrons is obtained from the hydrodynamic simulation (blue); red line shows a two-parameter low resolution fit. The black dashed line shows the laser temporal profile for comparison

The simulated temporal evolution of the total number of hot electrons is shown in Fig. 5 (blue) together with the corresponding laser profile (black). Up to about $+300\ {\rm{ps}}$ the hot electron production rate increases continuously through the laser pulse. There are noticeable maxima at about $+250\ {\rm{ps}}$ and $+500\ {\rm{ps}}$ as can be seen from a low resolution fit to the data (red). This indicates a significant temporal delay of the hot electron production with respect to the laser pulse. Such a delay is explained by a continuous increase of the plasma density scale length in the corona due to the plasma expansion. A similar temporal delay between the SRS back-reflected light with respect to the laser maximum has been observed in an experiment at the OMEGA laser facility^31^.

### Capacity of X-ray spectroscopy for suprathermal electron characterization

**Fig. 6:**
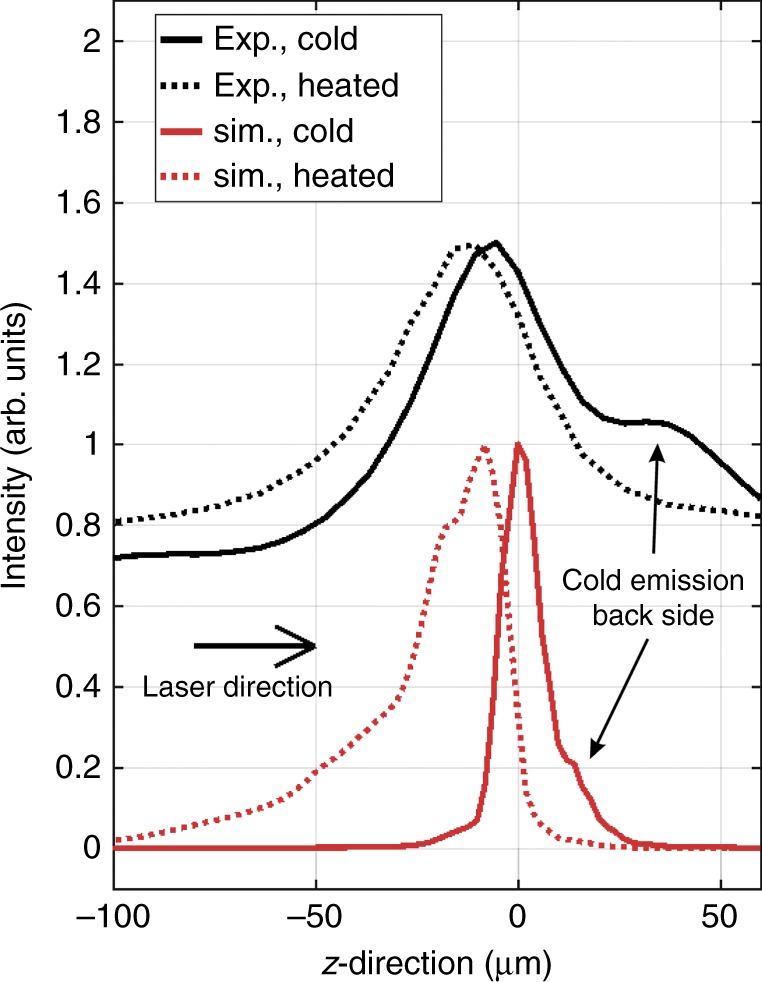
Spatial profiles of X-ray emission from plasma. Comparison of axial distributions of cold and heated ${\rm{K}}_{\alpha}$-emission obtained from the experiment and simulations

Figure 6 shows spatial profiles of the *S*1 emission group (solid lines, indicated as cold) compared to the emission of the groups $S2,S3,S4$ in the interval of 8120 to 8170 eV (dashed lines, indicated as heated). The emission peak of the heated lines is shifted about $10\ \upmu {\rm{m}}$ compared to the cold lines against the direction of the laser propagation in both experiment (black) and simulation (red). This is explained by the fact that the cold foil is moved forward while the heated plasma corona is accelerated and expanded towards the laser. The presence of the plasma corona explains extension of the heated emission, which has a similar distribution in the experiment and simulation (compare the spatial location of all emission groups in Figs. 2 and 3). Moreover, the simulation correctly shows the presence of an emission wing of the cold signal beyond the foil (indicated with an arrow in Fig. 6). Although qualitative agreement between the simulations and the measured data is evident, the data indicate a larger extension of cold foil emission in forward direction.

Figure 6 demonstrates the potential of the high-resolution X-ray spectroscopy and non-thermal atomic physics to provide critical data for benchmarking complex large scale hydrodynamic simulations that include non-linear plasma interactions.

### Effect of delay in hot electron production

**Fig. 7:**
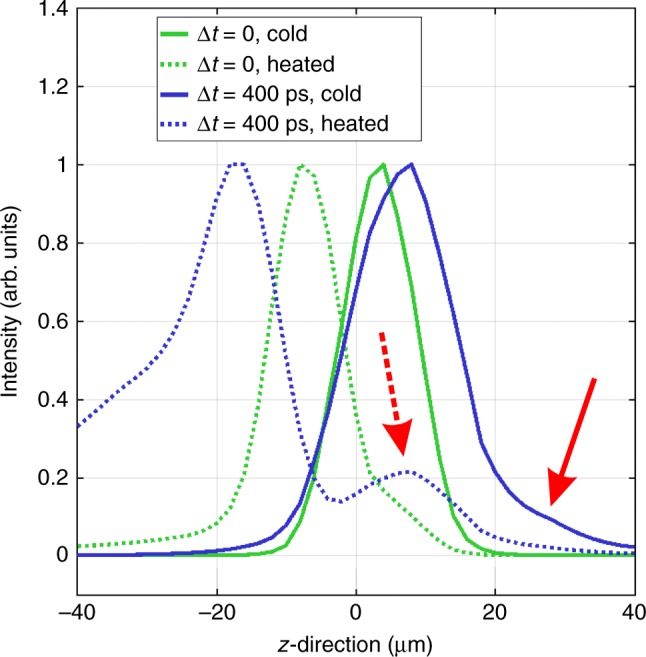
Effect of temporal delay on the X-ray emission. Simulated spatial distribution of K_α_-radiation emitted from cold and heated plasma regions. The electron distribution has a Gaussian profile delayed by $\Delta t=0$ or 400 ps versus the laser maximum

In order to discus the potential of high-resolution X-ray spectroscopy coupled to non-thermal atomic physics to validate complex large scale hydrodynamic simulations (that include non-linear plasma interactions driving suprathermal electrons via parametric instabilities) we consider two spectral simulations with hot electron temporal distributions artificially described by a Gaussian profile with FWHM = 250 ps and peak maxima at either 0 or +400 ps with respect to the laser maximum. The spatial profiles of the ${\rm{K}}_{\alpha}$-emission of both cold and heated regions are shown in Fig. 7 similarly as discussed in the previous sections. It is seen that if the laser and electron pulses are synchronized (green curves), the offset between the cold and heated emission is only about $10\ \upmu {\rm{m}}$, while in the case of $400\ {\rm{ps}}$ delayed hot electron population, this difference increases to about $30\ \upmu {\rm{m}}$. This increased shift is accompanied by the extended ${\rm{K}}_{\alpha}$-emission on the rear side due to the hydrodynamic evolution of the target (see red flashes in Fig. 7). Particularly, this combination of the emission maxima shift and the extended ${\rm{K}}_{\alpha}$-emission at the rear side of the cold expanding foil is indicative of relative time shifts of the suprathermal electron population with respect to the laser pulse. The high-resolution X-ray data coupled to non-thermal atomic physics has therefore a potential, as demonstrated in Fig. 6, to validate details of the non-linear laser plasma interaction in complex large scale hydrodynamic simulations.

### Effect of absolute number of hot electron population

**Fig. 8:**
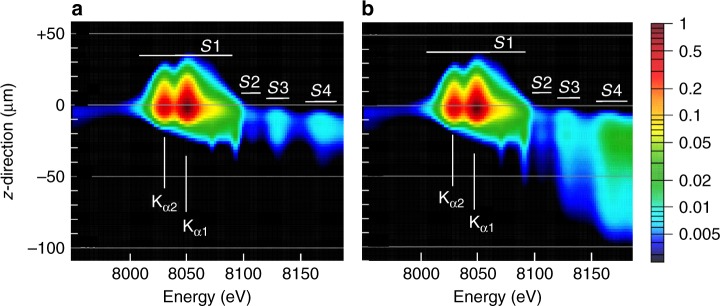
Effect of hot electron population. Synthetic spatially resolved spectra employing the same plasma parameters but different multiplication factors of the hot electron fraction, **a** hot electron populations from the ab initio simulation, **b** hot electron fraction decreased by a factor of 10

In order to demonstrate the sensitivity of the present non-thermal atomic physics analysis with respect to the hot electron fraction, Fig. 8 presents the synthetic spectrum with different number of hot electrons. Figure 8a presents simulations employing the ab initio suprathermal electron population shown in Fig. 5, while Fig. 8b shows the spectrum with the hot electron fraction artificially decreased by a factor of 10. Comparison of both figures shows that modification of the hot electron fraction has a large impact on the intensity and spatial evolution of ${\rm{K}}_{\alpha}$-emission groups $S2,S3,S4$. The enhanced emission of these groups relative to the ${\rm{K}}_{\alpha}$-emission in the spectral interval of 8000 to 8100 eV is explained as follows: The emission in the range 8000 to 8100 eV corresponds to low charged ions that are excited only by hot electrons. If the fraction of hot electrons decreases, the X-ray emission in this spectral range decreases accordingly, while the emission from higher charges states (groups $S2,S3,S4$) is unchanged because they are excited by bulk electrons. As the spectra in Fig. 8 are normalized to the X-ray emission of the group *S*1, the relative contribution of higher charge states in panel b is larger. This analysis demonstrates the potential of these emission groups to evaluate the absolute number of hot electrons and to validate corresponding predictions from large scale hydrodynamic simulations.

## Discussion

We have demonstrated a spectroscopic approach to measure the suprathermal electron evolution inside dense laser-heated plasma. Hot electrons are characterized via the detailed non-thermal atomic physics modeling of the spatially resolved X–ray emission based on the data obtained from large scale hydrodynamic simulations including non-linear plasma interactions. Our analysis shows very good overall qualitative agreement with the experiment and indicates a time delay of about $300\ {\rm{ps}}$ between the laser pulse maximum and the hot electron generation. The developed approach of coupling non-thermal atomic physics to highly spatially and spectrally resolved X-ray spectroscopic data and to hydrodynamic simulations that include parametric instabilities demonstrated its capacity for an advanced characterization of suprathermal electrons. These are of primary interest for inertial fusion and high energy density science. This approach can be further advanced in two ways. First, production and spatial distribution of hot electrons can be characterized via highly-resolved X-ray spectroscopic data coupled to non-thermal atomic physics. Alternatively, if the hydrodynamic motion and suprathermal electron population are known, the non-thermal atomic physics reconstruction serves as a powerful verification tool of the overall relevance of large-scale hydrodynamic codes that involve non-linear plasma interaction.

Concerning temporal evolution of suprathermal electrons (Fig. 5), the resonance absorption (being linear process depending on the plasma density scale length) may explain the second burst of electron production at about $+0.5$ ns, while the first maximum is due to the SRS. In the present hydrodynamic model each process of hot electron generation is characterized by the rate of hot electron production and their instantaneous effective temperature assuming an exponential distribution function in energy ${f}_{\rm{E}}\propto {\exp}(-E/{T}_{\mathrm{hot}})$. This model can be certainly improved by more detailed comparison with experiments and kinetic simulations on a microscopic level. X-ray spectroscopy is very sensitive to certain energy intervals, as ionization driven by hot electrons can only proceed if the electron energy exceeds the ionization potential. However, once the ionization threshold is reached, collisional processes show only a weak dependence on the parameter ${T}_{\mathrm{hot}}$ as demonstrated by Eq. (2). Therefore, in the atomic physics simulations, the hot electron fraction shown in Fig. 5 (red curve) was evaluated as the total number of hot electrons generated by all three processes (SRS, TPD and resonance absorption) with energy exceeding the Cu ionization potential of 9000 eV.

The atomic physics simulations demonstrate that the relative intensities of the ${\rm{K}}_{\alpha 1,2}$-lines (including the emission from charges states ${{\rm{Cu}}}^{+1}$ up to about ${{\rm{Cu}}}^{+15}$) and the emission groups $S1-S4$ are sensitive to the bulk electron temperature and the hot electron fraction. A comparison of the simulation results presented in Fig. 3 with the experimental data in Fig. 2 shows that the extended ${\rm{K}}_{\alpha}$-emission from the simulation agrees qualitatively with the observation and similarly all other ${\rm{K}}_\alpha$-emission features of groups $S1,S2,S3,S4$ are in qualitative agreement. The observed delayed hot electron generation is in good agreement with the evolution of the hot electron number from the simulations shown in Fig. 5. Figure 3 presents the first example of coupling the large scale non-linear laser-plasma hydrodynamic simulation to an atomic code modeling of the X-ray emission driven by hot electrons. The overall qualitative and reasonable quantitative agreement with the experimental data indicates that the hot electron model implemented in the hydrodynamic code^36^ has a predictive capacity.

## Methods

### Experimental setup

The experiment was performed at the PALS^39^ facility. The laser beam ($350\ {\rm{ps}}$, $700\ {\rm{J}}$, $1.315\ \upmu {\rm{m}}$) irradiated a $1.5-\upmu {\rm{m}}$–thick Cu foil at normal incidence. The laser beam profile and intensity distribution in the focal spot were characterized with calorimetric measurements of an attenuated beam. The spot profile was fit to a Gaussian function with $1/$e radius of $58.5\ \ \upmu {\rm{m}}$, and the energy encircled within the focal spot was carefully measured^38^. The maximum on-axis laser intensity, calculated using the measured laser temporal profile and spatial distribution, was found to be $4.55\times 1{0}^{15}\ {\rm{W}}\ {\rm{cm}}^{-2}$.

The Cu K–shell emission was observed by the Vertical geometry Johann Spectrometer (VJS) ^34^ equipped with a quartz (400) crystal cylindrically bent to a radius of 76 mm. This spectrometer provided simultaneously high-spatial resolution with $\delta z\approx 2\ \upmu {\rm{m}}$ (limiting intrinsic value) and high-spectral resolution of $\lambda /\Delta \lambda \approx 5200$ in a narrow spectral range $8000-8250\ {\rm{eV}}$ near the Cu ${\rm{K}}_\alpha$-group. The spectra were recorded on X–ray film Kodak Industrex AA400 and scanned with a pixel size of $5.3\ \upmu {\rm{m}}$. The signals were recalculated to intensities using known scanner and film calibration curves, crystal reflectivity, and filter transmissions. The experimental scheme is shown in Fig. 1, a spectral record from VJS is presented in Fig. 2.

### Coupled large-scale simulations

The theoretical model considers simultaneous and concurrent electron generation by the processes of resonant absorption, SRS and two-plasmon decay (TPD)^36^. Hot electron fluxes and temperatures are derived from the laser intensity in plasma calculated with the paraxial complex geometrical optics model coupled to the hydrodynamic code CHIC^37^ in the two-dimensional axially symmetric geometry. The simulation used the experimentally measured laser pulse energy, temporal and spatial profile. Examples of the spatial distribution of the plasma temperature and density for the times $t=0$ and $t=300\ {\rm{ps}}$ relative to laser maximum are shown in Fig. 4. Note, that the vertical bars of about $20\ \upmu {\rm{m}}$ thickness seen for large radii are related to the initialization of the cold material (the foil target outside of the focal spot has expanded to about $20\ \upmu {\rm{m}}$ due to simplified form of the equation of state used for the cold copper, this behavior does not influence the laser plasma interaction).

The CHIC hydrodynamic simulation provided also the number and mean energy of hot electrons generated by plasma instabilities with temporal and spatial resolution for the use in non-thermal atomic physics^20,25,26^.

The coupling of hydrodynamic simulations to atomic physics has been realized by interpolating the plasma and hot electron parameters from the CHIC non-structured Lagrangian mesh onto a regular Eulerian grid to calculate local quasi–stationary non–Maxwellian population kinetics and synthetic spectra by using the code FLYCHK^40^ at each time step. This approach is justified if the parameter ${n}_{\rm{e}}\tau$ (where $\tau$ is the ion confinement time) exceeds a critical value of $1{0}^{11}-1{0}^{12}\ {{\rm{cm}}}^{-3}{\rm{s}}$^20,26^. For an ion density of $0.1\ {\rm{g}}\ {\rm{cm}}^{-3}$ (${n}_{\rm{e}}\ \approx 1{0}^{22}\ {\rm{cm}}^{-3}$) in the deformed foil region as seen in Fig. 4b and a typical time scale of 100 ps, ${n}_{\rm{e}}\tau \approx 1{0}^{12}\ {{\rm{cm}}}^{-3}{\rm{s}}$. Relaxation effects are therefore expected to be small and the quasi-stationarity approach is valid.

### ${{\rm{K}}}_{\alpha }$ emission of copper ions

**Fig. 9:**
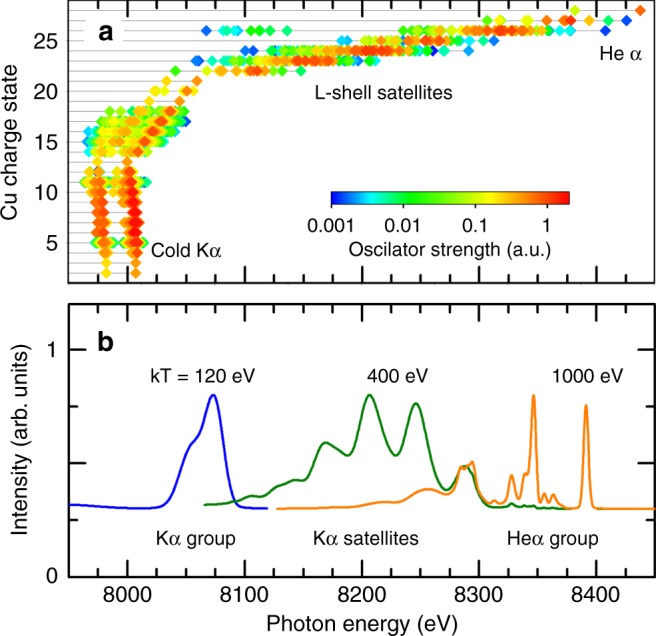
Atomic transitions. **a** Energies of K-shell transitions in various ionization states of Cu as calculated by the FAC code. **b** Synthetic single-cell spectra for different temperatures

The ionization states relevant for emission of the L-K transitions are presented in Fig. 9(a) as calculated by the FAC atomic code ^41^. It can be seen that numerous ${\rm{K}}_{\alpha}$-like transitions close to the original energy position of ${\rm{K}}_{\alpha 1,2}$-lines are emitted from charges states of ${{\rm{Cu}}}^{+1}$ …${{\rm{Cu}}}^{+15}$. The overlap of this emission leads to an effective broadening of the ${\rm{K}}_{\alpha 1,2}$-lines. For charge states up to ${{\rm{Cu}}}^{+15}$, the transition energies decrease with the increasing ionization and therefore correspond formally to a negative screening. The reasons for this anomalous behavior is essentially connected with the open $3d$-shell and also partially with the open $3p$-shell that imply a significant angular coupling and configuration interaction decreasing the transition energy. For the $3p$-shell, this phenomenon exists for the first ionizations of the $3p$-electrons but is successively overcompensated by the unscreened nuclear charge. Therefore for charge states higher than about $15$, transition energies increase along with the increasing ionization. Due to the photon energy overlap of so many charge states, a high-resolution X-ray spectroscopic analysis is mandatory to follow the temporal plasma evolution^42^. The strong emission of the group $S1$ in Fig. 2 is therefore essentially related to an overlap of the ${\rm{K}}_\alpha$-emissions from open M-shell configurations.

### Suprathermal electrons and the K-shell emission

Figure 9b visualizes the principle of reconstruction of the measured data. For the demonstration of the effect of the bulk plasma temperature ($T_{{\rm{bulk}}}$) on the simulated spectra a hot electron fraction $f_{\rm{hot}}=0.01 \%$, characteristic temperature $T_{\rm{hot}}=30\ {\rm{keV}}$ and total electron density ${n}_{\rm{e}}=1{0}^{22}\ {\rm{cm}}^{-3}$ was used. Here $f_{\rm{hot}}$ is defined as ^20,26^

1 $$f_{\rm{hot}}=\frac{{n}_{{\rm{e,hot}}}}{{n}_{{\rm{e,bulk}}}+{n}_{{\rm{e,hot}}}},$$

where $n_{{\rm{e,hot}}}$ and ${n}_{{\rm{e,bulk}}}$ are the densities of hot and bulk electrons. The observed emission is originating from radiative decay ${K}^{1}{L}^{x}{M}^{y}{N}^{z}\to {K}^{2}{L}^{x-1}{M}^{y}{N}^{z}+h\nu$ for different number of screening electrons in $L$-, $M$- and $N$-shell ($x$, $y$ and $z$ respectively). According to the electron configuration, three spectral regimes can be distinguished:

1. ${\rm{K}}_\alpha$-emission from singly ionized till F-like Cu ($x=8,y=0-18,z=0-1$); these transitions constitute a broad peak with photon energy between 8020 and 8100 eV (contributing to the emission group $S1$).
2. The ${\rm{K}}_\alpha$-satellite series, i.e., the emission from F– until Be– like Cu located in the spectral region 8100–8300 eV. These states differ in the number of electrons in the L shell ($x=3-8,y=z=0$) and contribute to the emission groups $S2,S3,S4$.
3. Thermal emission, i.e., the ${\rm{He}}_{\alpha}$-group with Li-like satellites ($x=2$, $y=z=0$, energy above $8300\ {\rm{eV}}$).

These regimes are clearly seen in the synthetic spectra, Fig. 9b: The spectrum for $T_{{\rm{bulk}}}=120\ {\rm{eV}}$ shows the cold ${\rm{K}}_{\alpha}$-group emission, however, it is already slightly shifted compared to the ${\rm{K}}_{\alpha 1}$-line due to M-shell ionization. For 400 eV, few of ${\rm{K}}_{\alpha}$-satellites are present, while for 1000 eV, the thermal ${\rm{He}}_\alpha$-emission dominates.

Production of the ${\rm{K}}_{\alpha}$-group and its satellites (energies below $8300\ {\rm{eV}}$) takes place in low temperature plasmas ($T_{\rm{bulk}}< \, 600\ {\rm{eV}}$) perturbed by hot electrons. The bulk electrons have too low energy to induce X–ray emission because the excitation and ionization rate coefficients $C$ and $I$ are proportional to $\exp (-\Delta E/T_{{\rm{bulk}}})/\sqrt{T_{{\rm{bulk}}}}$ where the transition energy $\Delta E$ is of the order of several keV. Therefore, an almost exponential cutoff takes place for temperatures much below 1 keV. The exponential factor is suppressed for hot electrons because $T_{\rm{hot}}\gg \Delta E$, providing

2 $${C}_{{\rm{hot}}},{I}_{{\rm{hot}}}\propto 1/\sqrt{T_{{\rm{hot}}}}.$$

This explains the origin of efficient ${\rm{K}}_{\alpha}$-emission in situations where the ionization degree is low due to low bulk electron temperatures. It is important to note that while the thermal part of emission is present in plasmas at temperatures around 1000–3000 eV, the two other ranges ($T \, < \, 600\ {\rm{eV}}$ in Fig. 9(b), blue and green curves) are practically absent in equilibrium plasmas. Their production in low temperature plasmas is due to hot electrons because the bulk electrons have too low temperature $T_{\rm{bulk}}$ that is insufficient to excite K-L transitions^20,43^.

### Streak record of plasma expansion

**Fig. 10:**
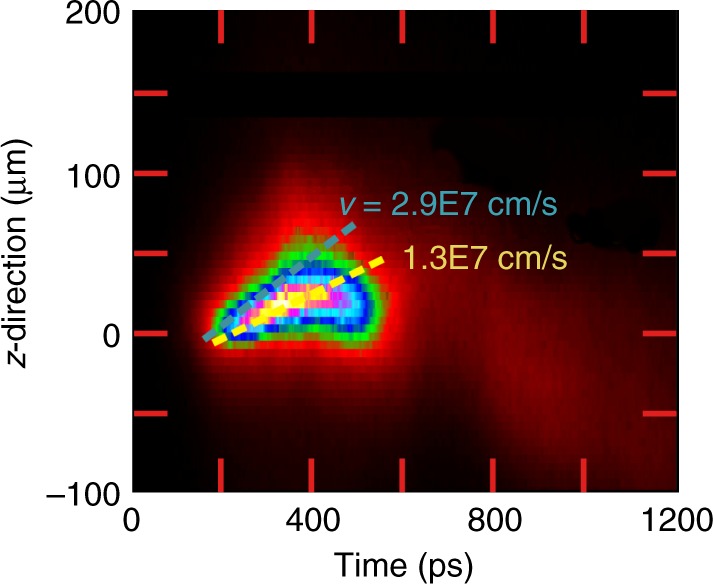
Plasma expansion visualized by X-ray streak camera. The laser is coming from below, the volume of the maximum emission moves with a velocity of $1.3\times 1{0}^{7}\ {\rm{cm}}/{\rm{s}}$

The streak camera was positioned perpendicular to the plasma expansion direction and only used for expansion velocity estimates derived from the plasma emission. Figure 10 shows an X-ray streak camera image of the plasma expansion with spatial and temporal resolution in the photon energy interval larger than 1 keV. Figure 10 clearly shows that the emission is moving in the direction of laser propagation (positive $z$-direction), the region with maximal intensity has a velocity of $1.3 \, \times 1{0}^{7}\ {\rm{cm}}/{\rm{s}}$. This value is in good agreement with the hydrodynamic simulation which has shown an offset of the dense foil of $30-50\ \upmu {\rm{m}}$ during $300\ {\rm{ps}}$ (see also Fig. 4) corresponding to an average velocity of about $1\!\!-\!\!1.7\times 1{0}^{7}\ {\rm{cm}}/{\rm{s}}$.

### Validation of simulations

The validation of large scale hydro simulations is a very complex and challenging problem under the conditions where non-linear processes play an important role. Validation of numerous approximations described in ref. ^36^ requires in the ideal case methods that are independent of laser-plasma simulations. As outlined above, this can be realized with atomic physics (non-Maxwellian atomic population kinetics, radiative properties) and high-resolution X-ray spectroscopy. Let us therefore discuss some particular critical issues in large scale hydrodynamic simulations and start with Fig. 4. The vertical bars near the surface structure observed for larger radii and extending to about $20\ \upmu {\rm{m}}$ are due to a limitation of the model related to the initialization of the cold material. Lagrangian methods are of limited use due to mesh distorsions which require mesh rezoning and remapping. Next, the thermal conduction model may overestimate lateral heat conduction. It requires the flux limitation or introduction of non-local heat transport models. This is important for the present case of thin targets and narrow focal spots where the electron mean free path may be comparable or larger than the local temperature scale lengths. Essential features for the present discussion (expansion velocity, temperature, and density evolution) calculated with the hydrodynamic code are in good agreement with atomic physics and spectroscopic calculations and measurements as shown in Fig. 3.

The time evolution of the total number of hot electrons as predicted by the hydrodynamic simulations show fine-scale temporal fluctuations (about 10 ps), cf. Fig. 5 (blue curve). These small-scale fluctuations are numerical artefacts that are related to too large hydrodynamic mesh size in the plasma corona that are not sufficient for fine resolution of plasma parameters near the threshold of parametric instabilities. Here, we are reaching the limits of the current modeling framework as the laser is rather intense, the spot size small, and the target thin. However, there are noticeable maxima at about $+250\ {\rm{ps}}$ and $+500\ {\rm{ps}}$ as can be seen from a low resolution fit to the data (red). This indicates a significant temporal delay of the hot electron population with respect to the laser pulse as indicated by the second maximum in Fig. 5 (red). Atomic physics is sensitive to these shifts as demonstrated in relation with Fig. 6.

The delayed ${\rm{K}}_\alpha$-emission could in principle be produced by hot-electron refluxing^32,33^. However, this is only important for relativistic electrons with a stopping range of $100\ \upmu {\rm{m}}$ or more. The present study concerns non-relativistic electrons with laser intensities below $1{0}^{16}\ {\rm{W}}\ {\rm{cm}}^{-2}$. The stopping range for the dominant part of hot electrons with energies <$20$ keV producing ${\rm{K}}_\alpha$-emission is of the order of $1\ \upmu {\rm{m}}$ and therefore below the target thickness. Refluxing is therefore negligible in the conditions considered in this paper.

## Data Availability

The data that support the findings of this study are available from authors upon request.
